# The Treatment of Yellow Fever

**Published:** 1878-09-20

**Authors:** 


### THE TREATMENT OF YELLOW FEVER.

---

[We are indebted, for the following article, to Dr. J. B. C. Gazzo, of La Fourche, La. It is translated from the French of the Rev. C. M. Menard, and appears of sufficient value to merit reproduction.—*Ed. Med. & Surg. Reporter.*]

The treatment is that of Dr. Cherot, of Martinique, and is said to have given the best results.—Ed. Record.

I.—FIRST TREATMENT.

Put the patient in bed; cover him well up to the neck; keep apartment closed. Without any delay, give patient a hot foot bath, strong with mustard, and leave feet in bath from eight to ten minutes; friction limbs, being particular not to let fresh air come in contact with patient; give two or three cups of elder-leaf tea, to produce perspiration; if convenient, place hot bricks, or bottles of hot water, around patient's legs. Apply mustard to feet for about one-quarter of an hour. Be very particular and not let patient uncover; this is all-important. As soon as possible, give patient lemonade every twenty minutes. The lemonade is made as follows: Two sour oranges (lemons will answer); peel, remove outer skin as much as possible, remove seeds, and boil six to ten minutes; let cool; sweeten, if desired, and administer. It is important to give this lemonade.

II.—DURING PERSPIRATION.

One hour after patient has taken to bed give clysters every three hours, followed by a foot bath without mustard. The clysters are prepared of mauve or gombo leaves, or of flax seed in small quantity. This treatment is kept up until the fever has subsided. Always be careful that patient is not exposed to air while perspiring. Continue to cause perspiration during four hours, and all the while give the above lemonade every twenty minutes for the first six hours, and then every thirty or forty minutes.

The patient must be made to perspire from four to five hours—not less than four hours. His clothes must be changed three, and (if corpulent and strong) four times. Warm the clothes well before changing, and while effecting a change do not let fresh air strike patient.

III.—AFTER PERSPIRATION.

When patient shall have well perspired from four to five hours, according to circumstances, remove covers, leaving only one sheet on patient if weather is warm, keeping the feet, however, well covered with a quilt or blanket. While giving clysters and foot baths keep apartment closed. If the weather is not cold or damp you may now open apartment so as to let a little fresh air circulate, being careful that patient is not in a current of air.

The fever lasts from twenty to forty hours, usually; sometimes, though rarely, from sixty to eighty hours. The pulse gives 100 to 120, sometimes 130 pulsations a minute. Do not be alarmed; keep patient in good spirits; keep him diverted, but do not fatigue him. Diversion contributes to a speedy cure.

Seven or eight hours after taking the fever the patient's tongue is spotted white, oftentimes black, on the centre, and is always encircled by a well-defined red girt.

IV.—WHEN THE FEVER CEASES.

As soon as the fever has subsided, and the pulse indicates sixty to seventy or eighty pulsations a minute—which happens at the end of about twenty hours—the patient must hasten to take, 1st, manna and senna, 2d, magnesia; 3d, peruvian bark and epsom salts; the *first* for ordinary cases; the *second* for weak patients, and the *third* for serious cases. These remedies are prepared and taken as follows:

1st. Manna and senna: for an adult, three ounces of manna well stirred in a cup of hot milk or water (the milk preferable); add an infusion of senna leaves; mix the whole together, strain, cool a little, and administer at a single dose, or in two doses. For a child, give one to one and a half ounce of manna, and but little of the senna infusion.

2d. Magnesia: three teaspoonfuls stirred in a cup of tepid water or milk. Give one dose.

3d. The most efficacious medicine of all: one ounce of pulverized peruvian bark (red) well stirred in a cupful of boiling water—stir and make a paste. In another cupful of boiling hot water, dissolve one-half ounce of epsom salts. Pour contents of both cups in an ordinary wine bottle, and fill the bottle threequarters full of hot water. Shake well and long, until the elbow tires. Let cool and give a little wineglassful every quarter of an hour. When patient begins to purge, give two little wineglassfuls every half hour. "This," said Dr. Cherot, "is *la medicine par excellence.*"

As soon as patient has taken above remedies, give him orange-leaf tea, or if that does not suit his palate, continue to give him the lemonade.

If medicine No. 1 (manna and senna) does not operate in two hours, give medicine No. 2 (magnesia). Be quick, for fear that fever will return or vomiting will occur. When these medicines are being taken, clysters followed by foot baths are discontinued.

When patient has had two operations, give him a little soup made of sour sorrel, the yolk of an egg, a little butter (preferable to lard), use very little salt, and soak in soup a thin slice of bread. This soup strengthens and does not interfere with taking of medicines. After five or six operations, begin with quinine, twenty to twenty-five grains given in five-grain doses every hour. For a child twelve to fifteen grains, in three-grain doses every hour.

If patient suffers too much from pain in the pit of the stomach, give him gum water—that is, gum arabic dissolved in hot water. Give one teaspoonful every now and then. One of the most critical stages of the disease is when the pain in the pit of the stomach increases. You must encourage patient; make him drink often. The disease apparently becomes more violent, the patient weakens rapidly, but he is unconsciously getting better.

The above is the treatment in ordinary cases.

V.—RETENTION OF URINE.

Too much care cannot be taken on this point. Press questions: if patient feels pain in the lower part of abdomen (a sure premonitory symptom of retention) friction the part every quarter of an hour, with camphorated oil. If retention prove obstinate, give pumpkin seed tea, or flaxseed tea, with one drachm of saltpetre for every five or six cups of the tea; and if patient is not perspiring give him a warm hip bath (not higher than the navel). Leave patient in hip bath one-quarter to one-half hour. It is highly important to remove retention.

VI.—VOMITING.

The vomiting of bile, water, and glaires, at the outset of the disease, is a good sign. Give tepid water to favor, but not enough to provoke vomiting.

Other vomiting of substance, bloody, and sometimes black, occurs in cases of relapse, or when fever has lasted too long, or because treatment has not been followed properly. Take peruvian bark pulverized (the red) one-half ounce, and put in an ordinary wine bottle three-quarters full of cold water. Shake well and long; let settle and give one tablespoonful every quarter of an hour and even oftener. Seven or eight doses usually suffice to check vomiting. After this, give the peruvian bark prepared with epsom salts as mentioned above. (See 3d, of No. IV.)

VII.—GANGRENE.

A thick, white and bloody coating of the inner mouth indicates the presence of gangrene, Administer the peruvian bark prepared with cold water (see No. VI) every quarter of an hour for half a day; then double the dose and give at intervals of half an hour, and finally every two hours. Purge patient every twenty-four or thirty-six hours, with the peruvian bark and epsom salts; then you may continue to give the peruvian bark with cold water, giving the doses further and further apart, until gangrene has disappeared.

Give emollient clyster, morning and night; administer the gum water; give a little soup; a little wine in a great deal of water—no pure wine—the least imprudence can cause death.

After giving the quinine, try and strengthen the patient with broth; a little wine in water—no cold water pure —nor pure wine; force patient to sit up and to get out of bed; do not expose him in current of air, nor let him go out in the damp or in the sun. Although he may not feel well, do not purge him unless in a grave case, because it is important not to overtax the stomach, already much enfeebled. If a little fever return and go off, give ten grains of quinine in two doses. Under no circumstances must you give pure cold water, even during the first three or four days of convalescence.

VIII.—SPECIAL OBSERVATIONS.

*Children* must be forced te drink lemonade and take medicines. If necessary stop up the nose and thus force them to drink. If threatened with attack of worms, give a little vermifuge. In all other respects treat them like adults, only give remedies in smaller doses.

*Women.*—If periodical complaint appear, that of itself is the best of remedies. In such a case, encourage flow by application of warm poultices on large veins inside of the thighs. Do not give lemonade and other remedies. Simply give orange leaf tea and the like harmless remedies. If the patient is one in a "delicate way," bolster head and stomach by means of pillows. No lemonade; orange leaf tea, but weak; foot baths, but not very warm; no clysters, except in serious cases, and then not frequent; manna, with not much senna, or simply magnesia, quinine, gum water; use great precaution during convalescence. Use same and greater precaution for women recently delivered,

IX.—IMPORTANT REMARKS.

1st. If patient throw up medicine or quinine, repeat the dose at onee. If patient be very sick at stomach, or swallow with difficulty, apply mustard on pit of the stomach for a few minutes, or take a bandage, say one inch wide, dip in cold water and apply under the chin, clear up to and under the ears.

2d. If patient does not eject first clyster at once, give a second, with Castile soap in it, and if that is not ejected, give a third, and then wait till the next regular time for giving clyster—say three hours.

3d. Stools are black and highly offensive to smell; are more difficult with women: you must act accordingly.

4th. Nursing must be attended to night and day, to see that patient is made to perspire well, to keep him covered, and to give him his medicines punctually.

5th. Except in rare instances, the disease lasts three days. If disease has not been properly attended to, vomiting and other serious symptoms usually occur on the third or fourth day.

6th. Relapse is dangerous, and is occasioned, 1st by exposing patient to air, or current of air; 2d, by his eating too much, of things indigestible; 3d, by laying in bed too long; 4th, by fatiguing patient, or allowing him to sit up late at night; 5th, by going out in the sun too soon.

7th. Convalescence is long and tedious, lasts from fifteen to twenty days. During convalescerse, give barley water, (*eau d'orge pele*).

Do not let patient fatigue; and do not allow him to go out in the sun for ten or twelve days.

Let him avoid excesses of all kinds.

